# *Cryptosporidium* spp. in large-scale sheep farms in China: prevalence and genetic diversity

**DOI:** 10.1038/s41598-024-62110-2

**Published:** 2024-05-16

**Authors:** Qianming Zhao, Meng Qi, Bo Jing, Fuchun Jian, Pihong Gong, Chenyang Lu, Yaqun Yan, Zhiyang Pei, Changshen Ning

**Affiliations:** 1 College of Veterinary Medicine, Henan Agricultural University, Zhengzhou, 450046 Henan People’s Republic of China; 2 College of Animal Science and Technology, Tarim University, Alar, 843300 Xinjiang People’s Republic of China; 3 School of Life Science and Agronomy, ZhouKou Normal University, Zhoukou, 466001 Henan People’s Republic of China

**Keywords:** *Cryptosporidium* spp., Large-scale sheep farms, Sheep, Feces, Environmental, Molecular epidemiology, China, Evolution, Microbiology, Molecular biology

## Abstract

*Cryptosporidium* spp. are significant zoonotic intestinal parasites that induce diarrhea and even death across most vertebrates, including humans. Previous studies showed that sheep are important hosts for *Cryptosporidium* and that its distribution in sheep is influenced by geography, feeding patterns, age, and season. Environmental factors also influence the transmission of *Cryptosporidium*. Molecular studies of *Cryptosporidium* in sheep have been conducted in only a few regions of China, and studies into the effect of sheep-housing environments on *Cryptosporidium* transmission are even rarer. To detect the prevalence of *Cryptosporidium* in large-scale sheep-housing farms, a total of 1241 fecal samples were collected from sheep, 727 environmental samples were taken from sheep housing, and 30 water samples were collected in six regions of China. To ascertain the existence of the parasite and identify the species of *Cryptosporidium* spp., we conducted nested PCR amplification of DNA extracted from all samples using the small-subunit (SSU) rRNA gene as a target. For a more in-depth analysis of *Cryptosporidium* spp. subtypes, *C. xiaoi*-and *C. ubiquitum*-positive samples underwent separate nested PCR amplification targeting the 60 kDa glycoprotein (*gp60*) gene. The amplification of the *Cryptosporidium* spp. SSU rRNA gene locus from the whole genomic DNA of all samples yielded a positive rate of 1.2% (20/1241) in fecal samples, 0.1% (1/727) in environmental samples, and no positive samples were found in water samples. The prevalence of *Cryptosporidium* spp. infection in large-scale housed sheep was 1.7%, which was higher than that in free-ranging sheep (0.0%). The highest prevalence of infection was found in weaning lambs (6.8%). Among the different seasons, the peaks were found in the fall and winter. The most prevalent species were *C. xiaoi* and *C. ubiquitum*, with the former accounting for the majority of infections. The distribution of *C. xiaoi* subtypes was diverse, with XXIIIc (n = 1), XXIIId (n = 2), XXIIIe (n = 2), and XXIIIl (n = 4) identified. In contrast, only one subtype, XIIa (n = 9), was found in *C. ubiquitum*. In this study, *C. xiaoi* and *C. ubiquitum* were found to be the predominant species, and *Cryptosporidium* was found to be present in the environment. These findings provide an important foundation for the comprehensive prevention and management of *Cryptosporidium* in intensively reared sheep. Furthermore, by elucidating the prevalence of *Cryptosporidium* in sheep and its potential role in environmental transmission, this study deepens our understanding of the intricate interactions between animal health, environmental contamination, and public health dynamics.

## Introduction

*Cryptosporidium* spp. are an intestinal parasite of medical and veterinary significance with the capacity to infect a wide range of vertebrates, including humans^1,2^. *Cryptosporidium* spp. infection are frequently asymptomatic, yet it has the potential to induce a range of gastrointestinal disorders in the host and can become life-threatening in severe instances^3^. An expert committee formed by the Food and Agriculture Organization of the United Nations (FAO) and the World Health Organization (WHO) identified *Cryptosporidium* spp. as one of the 24 most detrimental foodborne parasites^4^. *Cryptosporidium* spp. oocysts exhibit remarkable resilience, enabling them to retain their viability in water and other environmental media over extended durations. These oocysts are transmitted through direct contact with infected individuals (human-to-human transmission) or animals (zoonotic transmission), as well as by the consumption of contaminated food (foodborne transmission) and water (waterborne transmission)^5,6^.

Sheep are included among the primary hosts of *Cryptosporidium*, and the parasite is capable of inducing diarrhea and, in severe cases, mortality in lambs. This, in turn, leads to substantial economic losses for farmers^7^. *Cryptosporidium* infection rates in sheep vary from 0.9 to 76.9%. Approximately 46 distinct *Cryptosporidium* species have been identified, with 14 species displaying the capacity to infect sheep. Among these, *C. ubiquitum*, *C. xiaoi*, *C. andersoni,* and *C. parvum* are the most significant^8–10^. The types and occurrences of *Cryptosporidium* infections in sheep exhibit geographical diversity. Of particular concern for public health is the presence of *C. parvum* and *C. ubiquitum*, both prevalent in sheep, because of their association with Cryptosporidiosis^11^.

The sheep industry is a cornerstone of China’s animal husbandry sector, having experienced rapid growth in recent years. The most common rearing approach has transitioned from free-range grazing to large-scale-housed feeding, which mitigates the impact of external environmental conditions and seasonal fluctuations. While this shift has enhanced production efficiency, the elevated rearing density also creates an environment conducive to the occurrence of Cryptosporidiosis outbreaks^12^. There have been many studies on *Cryptosporidium* infections in sheep, but little is known about *Cryptosporidium* contamination in the living environment of housed sheep. In this study, we investigated *Cryptosporidium* infections and environmental contamination in large-scale-housed sheep farms by collecting fresh fecal samples from sheep as well as samples from the sheep's living environment. The results of the study help characterize the regional distribution of *Cryptosporidium* species and subtypes, which may be useful for the prevention and control of *Cryptosporidium* infections.

## Results

### Prevalence of *Cryptosporidium* species

In total, 1241 sheep fecal samples were gathered, of which 20 were positive for *Cryptosporidium*, yielding an infection rate of 1.6% (95% CI 0.9–2.3%). Within the six designated collection areas, *Cryptosporidium* infection prevalence among confined sheep in four regions was as follows: Henan, 1.5% (11/743); Ningxia, 1.9% (5/259); Jiangxi, 3.3% (2/60); and Tianjin, 6.1% (2/33). Notably, a significant difference in infection rates was observed between Tianjin and Henan (*P* < 0.05). A combined total of 727 environmental samples were gathered from Henan and Ningxia, with merely one sample (0.1%, 95% CI 0.0–0.4%) yielding a positive result. This sample originated from a large-scale sheep farm in Henan. Thirty water samples were collected and subjected to testing but yielded no positive readings for *Cryptosporidium* (Table 1).

**Table 1:** The prevalence and species distribution of *Cryptosporidium* spp. infection, as well as environmental contamination, among sheep across various regions.

Rearing system	Sample types	Sample area	Infection rate (% [no./total])	Species and subtypes (no.)
Scale housing	Fecal sample	Henan	1.5 (11/743)	*C. ubiquitum* (4), XIIa (4); *C. xiaoi* (6), XXIIIc (1), XXIIId (1), XXIIIe (1), XXIIIl (2); *C. parvum* (1)
		Ningxia	1.9 (5/259)	*C. ubiquitum* (4), XIIa (4); *C. andersoni* (1)
		Jiangxi	3.3 (2/60)	*C. xiaoi* (2), XXIIId (1), XXIIIl (1)
		Tianjin	6.1 (2/33)	*C. xiaoi* (1), XXIIIe (1); *C. ubiquitum* (1), XIIa (1)
		Heilongjiang	0.0 (0/30)	–
		Inner Mongolia	0.0 (0/28)	–
	Environments sample	Henan	0.2 (1/519)	*C. xiaoi* (1), XXIIIl (1)
		Ningxia	0.0 (0/195)	–
	Water sample	Henan	0.0 (0/30)	–
Free-range raising	Fecal sample	Ningxia	0.0 (0/88)	–
	Environmental sample	Ningxia	0.0 (0/13)	–
Total	Fecal sample		1.2 (20/1241)	*C. ubiquitum* (9), XIIa (9), *C. xiaoi* (9), XXIIIc (1), XXIIId (2), XXIIIe (2), XXIIIl (3); *C. parvum* (1); *C. andersoni* (1)
	Environmental sample		0.1 (1/727)	*C. xiaoi* (1), XXIIIl (1)
	Water sample		0.0 (0/30)	–

### *Cryptosporidium* infection in sheep at different physiological stages

Following the examination of 1127 fecal samples for the presence of *Cryptosporidium* in large-scale-housed sheep that were divided into nine well-defined physiological stages, the most remarkable occurrence of *Cryptosporidium* infection was in weaning lambs (6.8%, 95% CI 2.7–10.9%), which exhibited a considerably higher infection rate compared with fattening lambs (1.6%, 95% CI 0.0–3.5%) (*P* < 0.05). No instances of *Cryptosporidium* infection were identified among adult sheep, except for breeding rams, as shown in Table 2.

**Table 2:** Prevalence of *Cryptosporidium* spp. in large-scale housed sheep of different physiological stages.

Physiological state	Sample size	No. positive (%)	Species and subtypes (no.)
Lactating lambs	69	2 (2.9)	*C. xiaoi* (1), XXIIIc (1); *C. parvum* (1)
Weaning lambs	147	10 (6.8)	*C. xiaoi* (5), XXIIIe (2), XXIIIl (2); *C. ubiquitum* (5), XIIa (5)
Fattening lambs	184	3 (1.6)	*C. xiaoi* (3), XXIIId (2), XXIIIl (1)
Young lambs	142	3 (2.1)	*C. ubiquitum* (2), XIIa (2); *C. andersoni* (1)
Non-pregnant ewes	125	0 (0.0)	–
Early pregnancy ewes	111	0 (0.0)	–
Late pregnancy ewes	122	0 (0.0)	–
Lactating ewes	135	0 (0.0)	–
Breeding rams	92	2 (2.2)	*C. ubiquitum* (2), XIIa (2)
Total	1127	20 (1.8)	*C. ubiquitum* (9), XIIa (9); *C. xiaoi* (9), XXIIIc (1), XXIIId (2), XXIIIe (2), XXIIIl (3); *C. parvum* (1); *C. andersoni* (1)

### *Cryptosporidium* infection of sheep and contamination of the environment in different seasons

In the context of studying seasonal dynamics, we focused on Ruzhou-scale farms situated in Henan Province, China. The findings revealed that the infection rate reached its peak during winter (2.6%, 95% CI 0.0–2.2%) and hit its nadir in spring (0.9%, 95% CI 0–2.22%). Among the environmental samples, a single positive sample was identified during the summer, while the presence of *Cryptosporidium* was absent throughout the remaining seasons (Table 3).

**Table 3:** Prevalence of *Cryptosporidium* spp. in large-scale housed sheep and rates of positive environmental samples from different seasons.

Sample type	Sampling time	Infection rate (% [no./total])	Species and subtypes (no.)
Fecal sample	Spring	0.9 (2/222)	*C. ubiquitum* (2), XIIa (2)
	Summer	0.9 (1/106)	*C. xiaoi* (1), XXIIIc (1)
	Autumn	2.3 (3/129)	*C. ubiquitum* (1), XIIa (1); *C. xiaoi* (2), XXIIIl (2)
	Winter	2.6 (3/116)	*C. xiaoi* (3), XXIIId (1), XXIIIe (1)
Total		1.57 (9/573)	*C. ubiquitum* (3), XIIa (3); *C. xiaoi* (6), XXIIIc (1), XXIIId (1), XXIIIe (1), XXIIIl (1)
Environmental sample	Spring	0.0 (0/87)	
	Summer	0.9 (1/108)	*C. xiaoi* (1), XXIIIl (1)
	Autumn	0.0 (0/108)	
	Winter	0.0 (0/108)	
Total		0.2 (1/411)	*C. xiaoi* (1), XXIIIl (1)

### Genotyping and subtyping of *Cryptosporidium* spp. infecting sheep

The SSU rRNA gene fragments from the 21 *Cryptosporidium*-positive samples obtained in this experiment were effectively sequenced. The outcomes unveiled the existence of four distinct species: *C. xiaoi* (n = 10), *C. ubiquitum* (n = 9), *C. parvum* (n = 1), and *C. andersoni* (n = 1). For *C. xiaoi*, two sequences were procured, wherein six isolates exhibited complete congruence with a sequence identified in Algerian sheep (LC414392), while four isolates bore a resemblance to a sequence detected in Tibetan sheep (OL376597). In the case of *C. ubiquitum*, all nine isolates exhibited complete similarity to a sequence identified in British sheep (KM199742). A *C. parvum* isolate showed a complete resemblance to a sequence (OQ456120) identified in Chinese cows. Likewise, an isolate of *C. andersoni* exhibited complete congruity with a sequence (MK841325) detected in Chinese camels.

**Figure 1:**
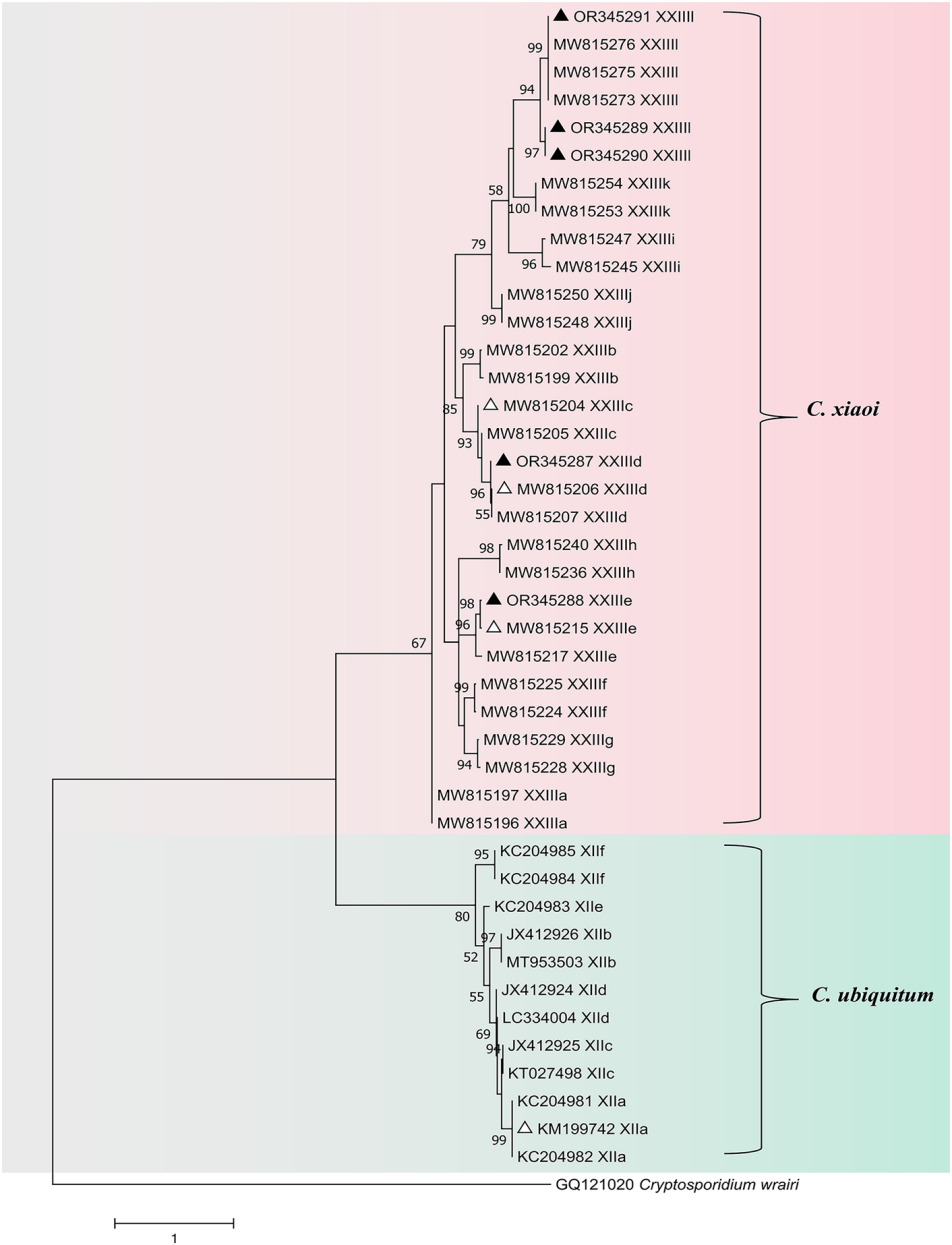
Molecular phylogenetic tree illustrating the genetic relationship between the *gp60* subtypes of *C. xiaoi* and *C. ubiquitum*. The evolutionary history was inferred using the maximum likelihood method, and evolutionary analyses were conducted in MEGA 7.0. Bootstrap values > 50% from 1000 replicates are shown on the nodes. The sequences detected in this study are shown with triangles; known sequences observed in this study are marked with open triangles, and new sequences are indicated by filled triangles.

The *gp60* gene was effectively amplified from nine isolates of *C. xiaoi* and an additional nine isolates of *C. ubiquitum*. Among these, *C. xiaoi* presented four distinct subtypes (XXIIIc, n = 1; XXIIId, n = 2; XXIIIe, n = 2; XXIIIl, n = 4), while *C. ubiquitum* exhibited a solitary subtype, XIIa. A phylogenetic tree was constructed to evaluate the genetic relationships between the *gp60* subtypes of *C. xiaoi* and *C. ubiquitum* (Fig. 1).

## Discussion

This study aimed to ascertain the prevalence of *Cryptosporidium* infections in large-scale-housed sheep and assess the presence of environmental contamination. Additionally, it was designed to evaluate the infection-related risk factors and identify the predominant species/genotypes of *Cryptosporidium* spp. in housed sheep. The average *Cryptosporidium* infection rate in this study was 1.6% (20/1241), which is generally lower than many other findings in China. For instance, Mi et al. reported a rate of 28.5% across 10 provinces in China, while Wu et al. observed a rate of 4.5% in Tibetan sheep from Gansu^7,13,14^. The overall infection rate in our study surpassed the 0.94% infection rate discovered in Xinjiang’s sheep in China by Qi et al. Furthermore, the results resembled Lang et al.’s findings for Inner Mongolian sheep (1.2%) and Penglin Wang et al.’s findings for sheep across eight provinces in China (1.9%)^9,15,16^. The results obtained in this study are also relatively low within the global context^10^. The variations in the aforementioned outcomes could potentially have stemmed from disparities in sampling locations, seasons, testing methodologies, feeding environments, and/or experimental frameworks.

*Cryptosporidium* is transmitted through the fecal–oral route, involving the ingestion of contaminated water or food, as well as animal-to-animal transmission. Among these, waterborne transmission is widely recognized as the primary mode of dissemination^17–20^. This study involved the collection of 714 environmental samples and 30 samples of sheep drinking water. Despite the identification of just a single positive sample for *Cryptosporidium* on the fecal leakage board among the environmental samples, this observation effectively underscores the existence of *Cryptosporidium* contamination of the housing of extensively barn-reared sheep in farming establishments. The reason that *Cryptosporidium* was not detected in environmental samples from other parts of the sheep barn may have been due to burial of the soil, resulting in *Cryptosporidium* oocysts not being collected at the time of sampling. *Cryptosporidium* was not detected in the water samples, a result that diverges from the findings of prior research^21^. This discrepancy might be linked to the utilization of sheep drinking bowls at this farm, which can diminish the introduction of impurities, like mud and dirt, by sheep while drinking, ultimately guaranteeing cleaner water. This cleaner drinking water, in turn, curbs pathogen transmission and diminishes the vulnerability of sheep to diseases.

In this study, the *Cryptosporidium* infection prevalence among large-scale-housed sheep (1.7%) surpassed that in free-ranging sheep (0.0%), aligning with findings from earlier research^16^. These outcomes could be attributed to several factors. Firstly, free-range sheep are spread across a broader expanse and thus experience limited inter-flock contact, consequently curtailing the likelihood of the disease spreading. Additionally, extensive rearing often integrates elevated intensification and commercialization, potentially subjecting sheep to persistent stress. This chronic stress might contribute to immune suppression, heightening the sheep’s vulnerability to pathogenic agents capable of inducing illness.

The majority of studies have consistently demonstrated that *Cryptosporidium* infection is more commonly observed in immature sheep than adult sheep^10,14,22^. This pattern was partially mirrored in the current study, as *Cryptosporidium* infections were absent in adult sheep, excluding the breeding rams. The high rate of infection in breeding rams may be due to the more fixed living enclosure and the contamination of the environment with *Cryptosporidium*, which can lead to persistent and repeated infections. It may also be related to the fact that breeding rams are involved in the task of mating, which leads to long-term stress and reduced immunity, increasing their risk of infection. The peak prevalence of *Cryptosporidium* infection (6.8%) surfaced among weaning lambs, which can be potentially attributed to three factors: (1) Post-weaning, lambs lose a source of immune factors from maternal milk, but their immune systems remain in the developmental stages, rendering them more vulnerable to pathogens^23^. (2) Following weaning, lambs commonly part from their mothers to initiate independent living, exposing them to environmental alterations that might provoke physical adjustments or maladaptation in the juveniles, thereby elevating disease susceptibility^24^. (3) After weaning, lambs undergo a significant change in their diet from mother’s milk to solid food. If the transition is inappropriate or the food is not suitable, it may lead to malnutrition, which in turn affects the functioning of the immune system.

In this study, we selected a large-scale-housed sheep farm in Ruzhou, Henan Province to investigate the seasonal dynamics of *Cryptosporidium* infection in sheep. When examining fecal samples, we observed a heightened infection prevalence during the autumn and winter seasons, in contrast to the lower prevalence during the spring and summer periods. This trend aligns with previous research outcomes^7^. This phenomenon could potentially have arisen from the heightened durability of *Cryptosporidium* oocysts in the cooler temperatures characteristic of autumn and winter^25,26^. Additionally, the inclination of sheep to huddle together for warmth in cooler temperatures might contribute to heightened contact and transmission rates. The reduced infection rates observed during spring (0.9%) and summer (0.9%) might be attributed to the elevated temperatures characteristic of the Ruzhou region in Henan Province, China, during these periods. Such temperatures may prove inhospitable to the ex vivo persistence of *Cryptosporidium* oocysts^27^. The seasonal dynamics of *Cryptosporidium*-contaminated environments were then investigated in this study. Only one positive sample for *Cryptosporidium* was detected in environmental samples collected from a manure leakage plate during the summer months. This may have been caused by the collection of samples that happened to come into contact with fresh sheep fecal samples containing *Cryptosporidium* oocysts.

*C. ubiquitum* is prevalent among ruminants, rodents, carnivores, and primates, encompassing approximately nine distinct genotypes (XIIa–XIIi)^28–31^. Despite *C. ubiquitum* exhibiting a wide host spectrum, certain subtypes display signs of host specificity. Notably, ruminants often contract subtype XIIa, which concurrently serves as the dominant subtype of *C. ubiquitum* in human infections^28^. In this investigation, we detected nine isolates of *C. ubiquitum* and determined that all nine were categorized under subtype XIIa at the *gp60* gene locus. This observation suggests that sheep could serve as significant intermediaries in the transmission of *C. ubiquitum* to humans, potentially facilitated by their direct interactions with infected animals or exposure to contaminated environments and drinking water sources.

*C. xiaoi* is usually found in sheep and goats and is the dominant *Cryptosporidium* species in sheep in many parts of China^7,32^ as well as the United States and Australia^33,34^. There are 12 subtypes (XXIIIa–XXIIIl) currently identified^35^. In this research, nine isolates of *C. xiaoi* were effectively subjected to subtyping, revealing the presence of four distinct subtypes: XXIIIc (n = 1), XXIIId (n = 2), XXIIIe (n = 2), and XXIIIl (n = 4). Yingying Fan et al. did not detect sheep infections involving XXIIIc; however, this study’s outcomes disclosed the presence of *C. xiaoi* XXIIIc infection in sheep^35^. These findings align with Lang et al.’s observations in Inner Mongolia^16^, effectively broadening the known spectrum of hosts susceptible to *C. xiaoi* XXIIIc.

*C. parvum* and *C. andersoni* constitute the predominant species of *Cryptosporidium* known to infect humans^36^. However, it is worth noting that *C. andersoni* predominantly targets primates and equids, whereas *C. parvum* displays a considerably wider spectrum of hosts, encompassing primates, ruminants, equids, and rodents^37^. Therefore, zoonotic Cryptosporidiosis is mainly caused by *C. parvum*. Simultaneously, the increasing occurrence of *C. andersoni* in humans is contributing to an incremental escalation in its potential as a public health hazard^38–41^. While this study unearthed merely one positive sample for each of *C. parvum* and *C. andersoni*, it is essential to remain vigilant to the potential public health and safety complications that might arise from outbreaks involving these two *Cryptosporidium* species.

## Conclusions

This study determined that there was a cumulative *Cryptosporidium* infection rate of 1.7% (20/1153) in large-scale-housed sheep across certain regions of China. Moreover, four distinct *Cryptosporidium* species were discovered infecting the sheep: *C. xiaoi*, *C. ubiquitum*, *C. parvum*, and *C. andersoni*. Notably, *C. xiaoi* and *C. ubiquitum* emerged as the prevailing species. A singular instance of *Cryptosporidium* positivity was identified in an environmental sample, signifying the existence of *Cryptosporidium*-associated environmental contamination. *Cryptosporidium* was not detected in water samples. These findings establish a crucial foundation for the comprehensive prevention and management of *Cryptosporidium* in intensively reared sheep. Furthermore, by elucidating the prevalence of *Cryptosporidium* in sheep and its potential role in environmental transmission, this study enhances our comprehension of the intricate interplay among animal health, environmental pollution, and public health dynamics.

## Materials and methods

### Fecal sample collection

Between March 2021 and March 2023, a total of 1,998 samples were gathered from six distinct regions across China: Henan, Ningxia, Heilongjiang, Jiangxi, Inner Mongolia, and Tianjin. This comprehensive compilation included 1,241 sheep fecal samples (1153 from large-scale-housed sheep farms and 88 from free-range farms), 727 samples from the sheep living environments (714 from large-scale-housed sheep farms and 13 from free-range farms), and 30 water samples collected in a large-scale-housed sheep farm situated in Henan Province. These details are presented in Table 1.

Fecal samples were collected from sheep at nine physiological and developmental stages: lactating lambs, weaned lambs, fattening lambs, young sheep, empty ewes, pre-pregnant ewes, post-pregnant ewes, lactating ewes, breeding rams. Samples of 5–30 g of feces were collected rectally one at a time, placed in a clean plastic bag, numbered, registered, returned to the laboratory, and stored in a refrigerator at 4 °C for examination.

### Collection of environmental samples

Environmental samples of 5–30 g were randomly collected at the different physiological stages sheep house entrance, fecal leakage board, sheep house exit, and corridor. The samples were placed in clean plastic bags, numbered, registered, and returned to the laboratory to be refrigerated (4 °C) before testing.

### Water sample collection

A Ruzhou-scale farm in Henan Province was selected, and 2–4 water samples were collected from enclosures of sheep at different physiological stages, with a total of 30 samples collected. Each sample collected was 50 mL, which was placed in a clean centrifuge tube, numbered, registered, and brought back to the laboratory to be stored in the refrigerator at 4 °C before examination.

### Seasonal dynamic survey of scale farms

To assess for sheep digestive tract infections and environmental contamination of *Cryptosporidium* spp. across various seasons within large-scale farms, we gathered samples from farms in Ruzhou City every quarter, resulting in a total of 573 fecal samples and 411 environmental samples. The samples were placed in clean plastic bags, numbered, registered, and returned to the laboratory to be refrigerated (4 °C) before testing.

### DNA extraction and PCR amplification

DNA extraction from fecal and environmental samples involved placing approximately 100 mg of each sample into a 1.5 ml centrifuge tube. The E.Z.N.A. Stool DNA Kit (Omega Bio-Tek Inc., Norcross, GA, USA) was utilized for whole-genome DNA extraction. Subsequently, 200 μL of DNA extract was collected, labeled, and stored at − 20 °C before subsequent testing.

DNA was extracted from the water samples as follows: a centrifuge tube containing 50 mL of water sample underwent centrifugation at 3500 rpm for 10 min. The supernatant was then discarded, leaving the precipitate. The DNeasy PowerSoil Pro kit (QIAGEN Inc., Hilden, Germany) was employed to extract the entire genomic DNA from the precipitate. Subsequently, 50 μL of DNA extract was collected, labeled, and stored at − 20 °C.

To ascertain the existence of the parasite and identify the species of *Cryptosporidium* spp., we conducted nested PCR amplification of DNA extracted from all samples using the small-subunit (SSU) rRNA gene as a target^42^. For a more in-depth analysis of *Cryptosporidium* spp. species and subtypes, *C. xiaoi-* and *C. ubiquitum*-positive samples underwent separate nested PCR amplification targeting the 60 kDa glycoprotein (*gp60*) gene^28,35^. The amplified products underwent 1% agarose gel electrophoresis using DNA Green dye for visualization, and the analysis was conducted using a Tanon 3500 gel image analysis system.

### Sequencing and phylogenetic analyses

Secondary positive amplified fragments were forwarded to either Beijing SinoGenoMax Co. Ltd. or Sangon Bioengineering Co. (Shanghai) for purification and bi-directional sequencing. The BLAST program (http://blast.ncbi.nlm.nih.gov/Blast.cgi) was employed to analyze all sequences through homology searches. Representative nucleotide sequences obtained during this study have been deposited in the GenBank database and assigned accession numbers. The accession number for the *gp60* gene is OR345287–OR345291.

### Statistical analysis

Statistical analysis was carried out using SPSS version 22.0 (SPSS Inc.) and utilizing a chi-square test with a 95% confidence interval. This analysis was conducted to compare *Cryptosporidium* spp. infection rates across distinct collection sites, physiological stages, and seasons. A significance level of *P* value < 0.05 was employed to determine the statistical significance of the differences between groups.

### Ethics approval and consent to participate

The study was conducted in accordance with the Chinese Law on the Management of Laboratory Animals (1988) and was reviewed and approved by the Research Ethics Committee of Henan Agricultural University. Permission was obtained from the farm owner before faecal samples were collected. In this study, all faecal samples were carefully collected from the rectum of each dairy cattle without causing harm.

## Data Availability

The datasets supporting the conclusions of this article are included within the article. Representative sequences are submitted to the GenBank database under the following accession numbers: OR345287–OR345291.

## Abbreviations

C.: *Cryptosporidium*
Spp.: Species
PCR: Polymerase Chain Reaction
SSU: Small-subunit
*gp60*: 60 KDa glycoprotein
